# Re: Vitamin D supplementation for improving sperm parameters in infertile men: A systematic review and meta-analysis of randomized clinical trials

**DOI:** 10.1080/2090598X.2023.2204459

**Published:** 2023-04-25

**Authors:** Ratih Rinendyaputri, Uly Alfi Nikmah, Sela Septima Maria

**Affiliations:** Biomedical Research Center, Health Research Organization, National Research and Innovation Agency (BRIN), Cibinong, Indonesia

Dear Editor,

We are grateful for the systematic review and meta-analysis on the effects of vitamin D in infertile patients [1]. Various journals have reported the success and mechanisms of vitamin D in the treatment of infertile patients. With this systematic review and meta-analysis, it can be used as a clinical consideration in providing vitamin D therapy to infertile patients.

In this systematic review and meta-analysis, it concludes that “Vitamin D supplementation may improve sperm motility, progressive sperm motility, and morphology in infertile men“. This is supported by several journals that vitamin D acts as an antioxidant against oxidative stress on the sperm membrane which can damage DNA. This is indicated by a decrease in 4-Hydroxynonenal levels due to the administration of vitamin D, so it has an effect on sperm count, motility and morphology [2]. Vitamin D can affect the hormone testosterone which is also influenced by age and nutrition, so that this variation greatly influences the success of vitamin D therapy in infertile men [3]. Positive correlation of vitamin D in serum and its effect in vitro has been shown to enhance sperm motility [4]. In infertile patients who have a deficiency of vitamin D in the blood serum affects sperm motility and morphology [5].

For clinicians, this can be a reference but need to be noted in Table 2 and Figure 2. First, in the characteristics table 2, it shows that the population have variations in vitamin D doses, age and infertility diagnoses even though it has low risk in all eligible articles. Second, on subgroups (Figure 2) in the total sperm count, progressive sperm motility and sperm morphology subgroup had an I^2^ (>75%) that indicates the heterogeneity of the data. Although the conclusion of this article shows that there is an effect of Vitamin D which has a positive effect on sperm motility, progressive sperm motility and morphology in infertility, the heterogeneity of the data needs to be considered in making conclusions.
